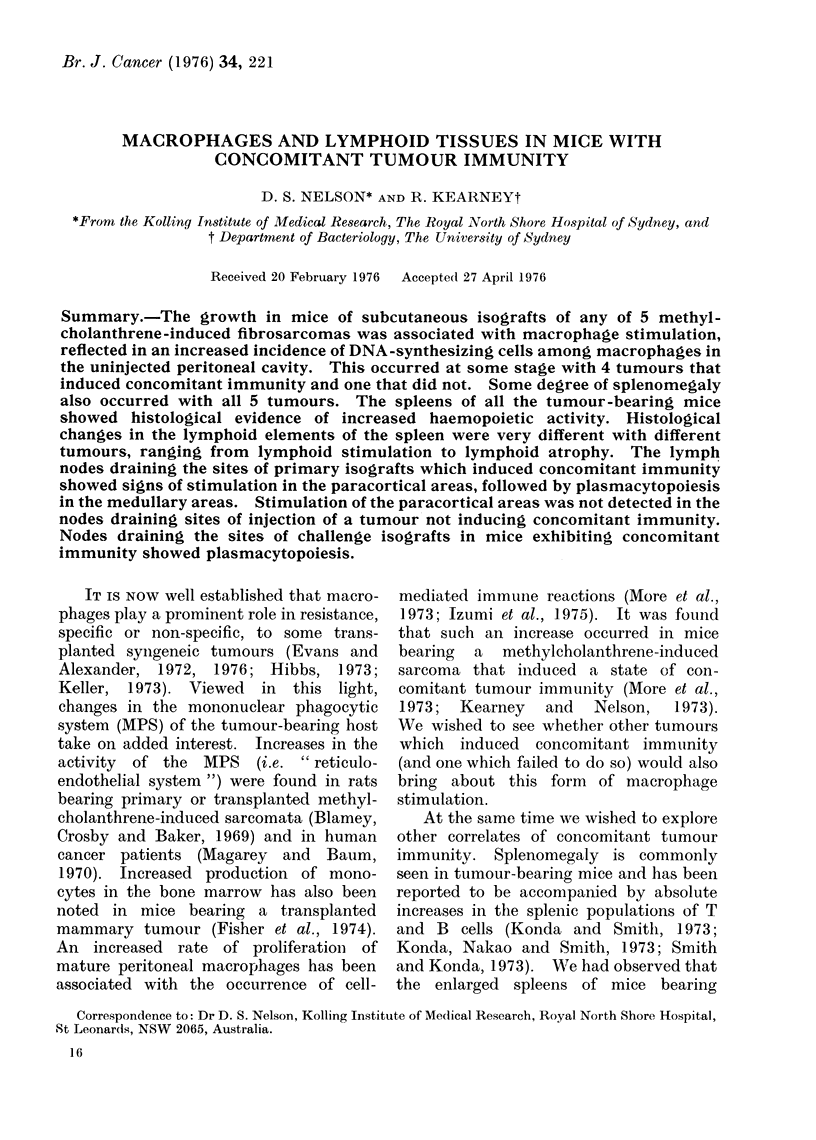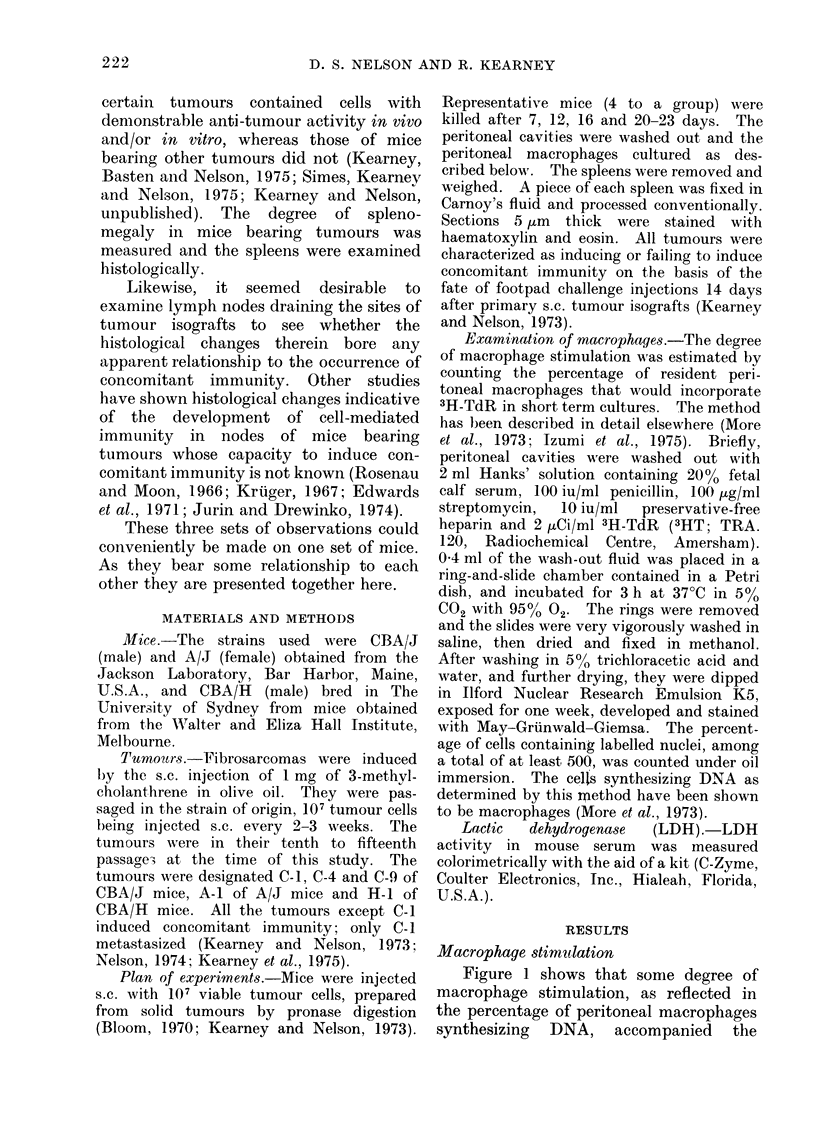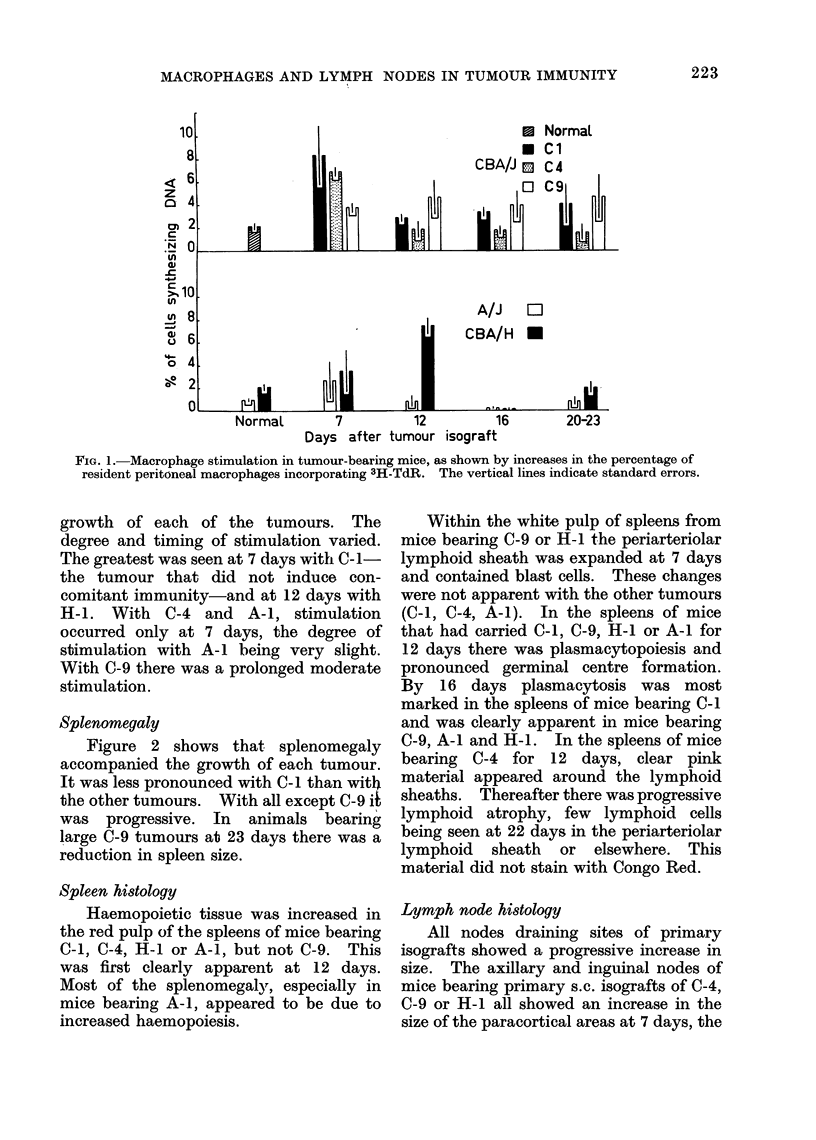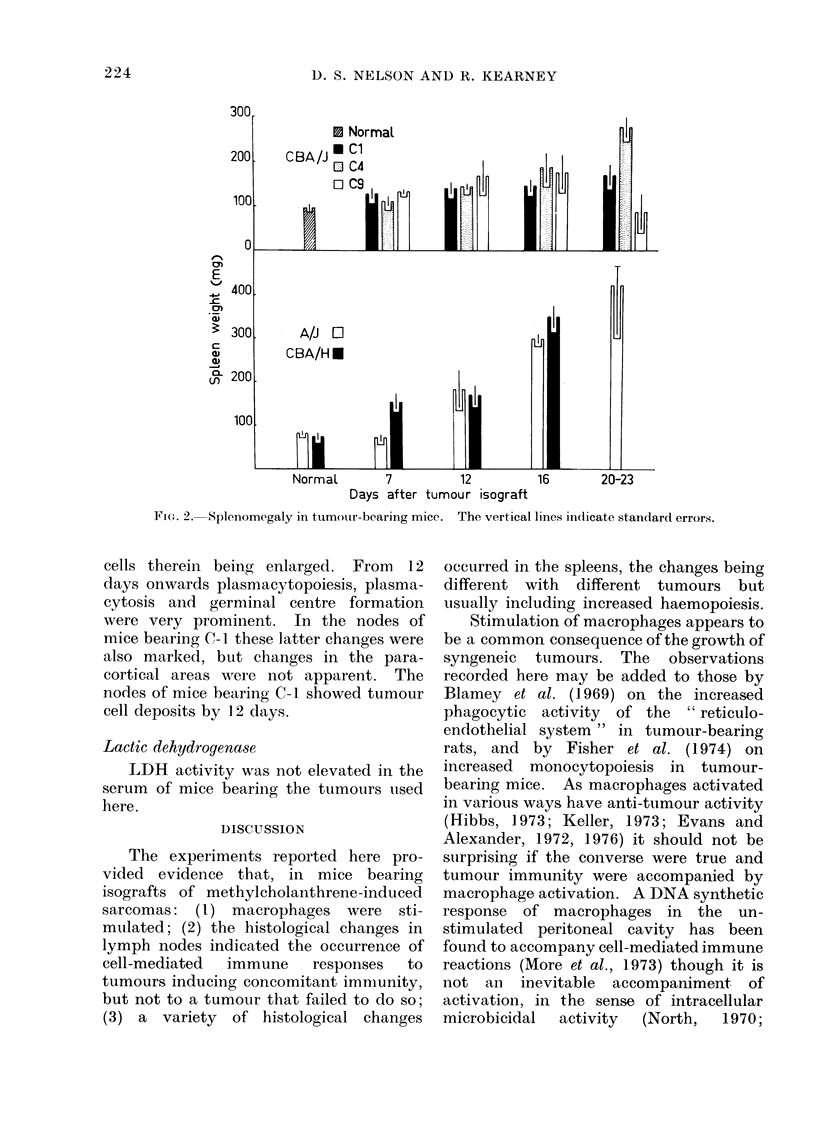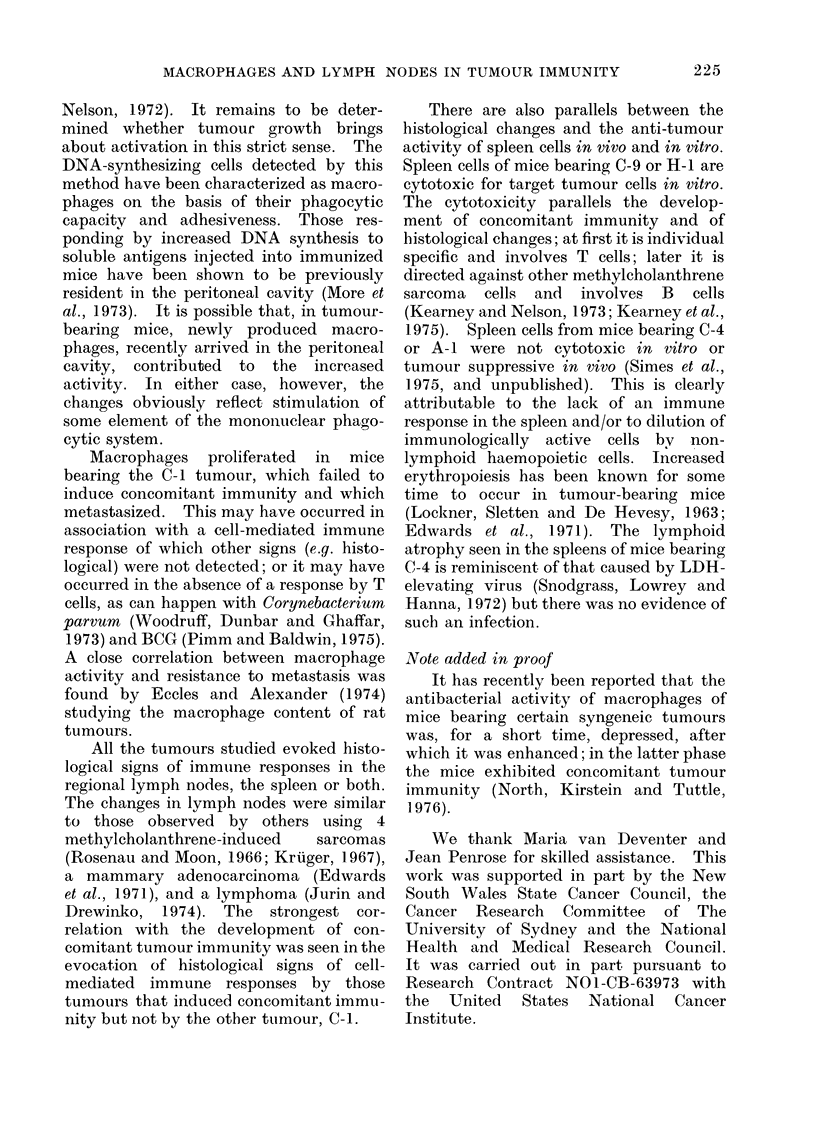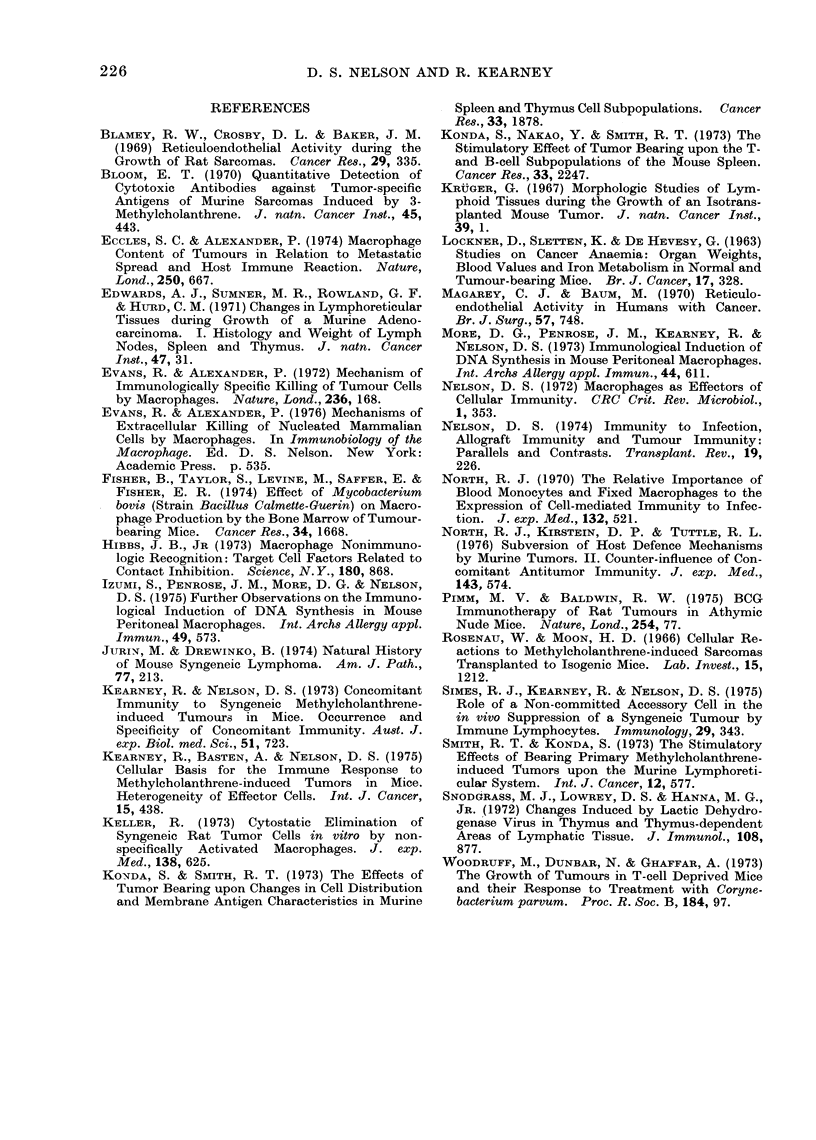# Macrophages and lymphoid tissues in mice with concomitant tumour immunity.

**DOI:** 10.1038/bjc.1976.155

**Published:** 1976-09

**Authors:** D. S. Nelson, R. Kearney

## Abstract

The growth in mice of subcutaneous isografts of any of 5 methylcholanthrene-induced fibrosarcomas was associated with macrophage stimulation, reflected in an increased incidence of DNA-synthesizing cells among marcophages in the uninjected peritoneal cavity. This occurred at some stage with 4 tumours that induced concomitant immunity and one that did not. Some degree of splenomegaly also occurred with all 5 tumours. The spleens of all the tumour-bearing mice showed histological evidence of increased haemopoietic activity. Histological changes in the lymphoid elements of the spleen were very different with different tumours, ranging from lymphoid stimulation to lymphoid atrophy. The lymph nodes draining the sites of primary isografts which induced concomitant immunity showed signs of stimulation in the paracortical areas, followed by plasmacytopoiesis in the medullary areas. Stimulation of the paracortical areas was not detected in the nodes draining sites of injection of a tumour not inducing concomitant immunity. Nodes draining the sites of challenge isografts in mice exhibiting concomitant immunity showed plasmacytopiesis.


*Br. J. Cancer* (1976) **34**, 221

### MACROPHAGES AND LYMPHOID TISSUES IN MICE WITH CONCOMITANT TUMOUR IMMUNITY

D. S. NELSON* AND R. KEARNEY†

*From the Kolling Institute of Medical Research, The Royal North Shore Hospital of Sydney, and
† Department of Bacteriology, The University of Sydney*

Received 20 February 1976 Accepted 27 April 1976

**Summary.—The growth in mice of subcutaneous isografts of any of 5 methylcholanthrene-induced fibrosarcomas was associated with macrophage stimulation, reflected in an increased incidence of DNA-synthesizing cells among macrophages in the uninjected peritoneal cavity. This occurred at some stage with 4 tumours that induced concomitant immunity and one that did not. Some degree of splenomegaly also occurred with all 5 tumours. The spleens of all the tumour-bearing mice showed histological evidence of increased haemopoietic activity. Histological changes in the lymphoid elements of the spleen were very different with different tumours, ranging from lymphoid stimulation to lymphoid atrophy. The lymph nodes draining the sites of primary isografts which induced concomitant immunity showed signs of stimulation in the paracortical areas, followed by plasmacytopoiesis in the medullary areas. Stimulation of the paracortical areas was not detected in the nodes draining sites of injection of a tumour not inducing concomitant immunity. Nodes draining the sites of challenge isografts in mice exhibiting concomitant immunity showed plasmacytopoiesis.**

IT IS NOW well established that macrophages play a prominent role in resistance, specific or non-specific, to some transplanted syngeneic tumours (Evans and Alexander, 1972, 1976; Hibbs, 1973; Keller, 1973). Viewed in this light, changes in the mononuclear phagocytic system (MPS) of the tumour-bearing host take on added interest. Increases in the activity of the MPS (*i.e.* " reticulo-endothelial system ") were found in rats bearing primary or transplanted methylcholanthrene-induced sarcomata (Blamey, Crosby and Baker, 1969) and in human cancer patients (Magarey and Baum, 1970). Increased production of monocytes in the bone marrow has also been noted in mice bearing a transplanted mammary tumour (Fisher *et al.*, 1974). An increased rate of proliferation of mature peritoneal macrophages has been associated with the occurrence of cell-mediated immune reactions (More *et al.*, 1973; Izumi *et al.*, 1975). It was found that such an increase occurred in mice bearing a methylcholanthrene-induced sarcoma that induced a state of concomitant tumour immunity (More *et al.*, 1973; Kearney and Nelson, 1973). We wished to see whether other tumours which induced concomitant immunity (and one which failed to do so) would also bring about this form of macrophage stimulation.

At the same time we wished to explore other correlates of concomitant tumour immunity. Splenomegaly is commonly seen in tumour-bearing mice and has been reported to be accompanied by absolute increases in the splenic populations of T and B cells (Konda and Smith, 1973; Konda, Nakao and Smith, 1973; Smith and Konda, 1973). We had observed that the enlarged spleens of mice bearing

Correspondence to: Dr D. S. Nelson, Kolling Institute of Medical Research, Royal North Shore Hospital, St Leonards, NSW 2065, Australia.

certain tumours contained cells with demonstrable anti-tumour activity *in vivo* and/or *in vitro,* whereas those of mice bearing other tumours did not (Kearney, Basten and Nelson, 1975; Simes, Kearney and Nelson, 1975; Kearney and Nelson, unpublished). The degree of splenomegaly in mice bearing tumours was measured and the spleens were examined histologically.

Likewise, it seemed desirable to examine lymph nodes draining the sites of tumour isografts to see whether the histological changes therein bore any apparent relationship to the occurrence of concomitant immunity. Other studies have shown histological changes indicative of the development of cell-mediated immunity in nodes of mice bearing tumours whose capacity to induce concomitant immunity is not known (Rosenau and Moon, 1966; Krüger, 1967; Edwards *et al.*, 1971; Jurin and Drewinko, 1974).

These three sets of observations could conveniently be made on one set of mice. As they bear some relationship to each other they are presented together here.

### MATERIALS AND METHODS

*Mice.*—The strains used were CBA/J (male) and A/J (female) obtained from the Jackson Laboratory, Bar Harbor, Maine, U.S.A., and CBA/H (male) bred in The University of Sydney from mice obtained from the Walter and Eliza Hall Institute, Melbourne.

*Tumours.*—Fibrosarcomas were induced by the s.c. injection of 1 mg of 3-methylcholanthrene in olive oil. They were passaged in the strain of origin, $10^7$ tumour cells being injected s.c. every 2–3 weeks. The tumours were in their tenth to fifteenth passages at the time of this study. The tumours were designated C-1, C-4 and C-9 of CBA/J mice, A-1 of A/J mice and H-1 of CBA/H mice. All the tumours except C-1 induced concomitant immunity; only C-1 metastasized (Kearney and Nelson, 1973; Nelson, 1974; Kearney *et al.*, 1975).

*Plan of experiments.*—Mice were injected s.c. with $10^7$ viable tumour cells, prepared from solid tumours by pronase digestion (Bloom, 1970; Kearney and Nelson, 1973). Representative mice (4 to a group) were killed after 7, 12, 16 and 20–23 days. The peritoneal cavities were washed out and the peritoneal macrophages cultured as described below. The spleens were removed and weighed. A piece of each spleen was fixed in Carnoy's fluid and processed conventionally. Sections 5 μm thick were stained with haematoxylin and eosin. All tumours were characterized as inducing or failing to induce concomitant immunity on the basis of the fate of footpad challenge injections 14 days after primary s.c. tumour isografts (Kearney and Nelson, 1973).

*Examination of macrophages.*—The degree of macrophage stimulation was estimated by counting the percentage of resident peritoneal macrophages that would incorporate $^3$H-TdR in short term cultures. The method has been described in detail elsewhere (More *et al.*, 1973; Izumi *et al.*, 1975). Briefly, peritoneal cavities were washed out with 2 ml Hanks' solution containing 20% fetal calf serum, 100 iu/ml penicillin, 100 μg/ml streptomycin, 10 iu/ml preservative-free heparin and 2 μCi/ml $^3$H-TdR ($^3$HT; TRA. 120, Radiochemical Centre, Amersham). 0·4 ml of the wash-out fluid was placed in a ring-and-slide chamber contained in a Petri dish, and incubated for 3 h at 37°C in 5% $CO_2$ with 95% $O_2$. The rings were removed and the slides were very vigorously washed in saline, then dried and fixed in methanol. After washing in 5% trichloracetic acid and water, and further drying, they were dipped in Ilford Nuclear Research Emulsion K5, exposed for one week, developed and stained with May–Grünwald–Giemsa. The percentage of cells containing labelled nuclei, among a total of at least 500, was counted under oil immersion. The cells synthesizing DNA as determined by this method have been shown to be macrophages (More *et al.*, 1973).

*Lactic dehydrogenase* (LDH).—LDH activity in mouse serum was measured colorimetrically with the aid of a kit (C-Zyme, Coulter Electronics, Inc., Hialeah, Florida, U.S.A.).

### RESULTS

### *Macrophage stimulation*

Figure 1 shows that some degree of macrophage stimulation, as reflected in the percentage of peritoneal macrophages synthesizing DNA, accompanied the

FIG. 1.—Macrophage stimulation in tumour-bearing mice, as shown by increases in the percentage of resident peritoneal macrophages incorporating $^3$H-TdR. The vertical lines indicate standard errors.

growth of each of the tumours. The degree and timing of stimulation varied. The greatest was seen at 7 days with C-1—the tumour that did not induce concomitant immunity—and at 12 days with H-1. With C-4 and A-1, stimulation occurred only at 7 days, the degree of stimulation with A-1 being very slight. With C-9 there was a prolonged moderate stimulation.

*Splenomegaly*

Figure 2 shows that splenomegaly accompanied the growth of each tumour. It was less pronounced with C-1 than with the other tumours. With all except C-9 it was progressive. In animals bearing large C-9 tumours at 23 days there was a reduction in spleen size.

*Spleen histology*

Haemopoietic tissue was increased in the red pulp of the spleens of mice bearing C-1, C-4, H-1 or A-1, but not C-9. This was first clearly apparent at 12 days. Most of the splenomegaly, especially in mice bearing A-1, appeared to be due to increased haemopoiesis.

Within the white pulp of spleens from mice bearing C-9 or H-1 the periarteriolar lymphoid sheath was expanded at 7 days and contained blast cells. These changes were not apparent with the other tumours (C-1, C-4, A-1). In the spleens of mice that had carried C-1, C-9, H-1 or A-1 for 12 days there was plasmacytopoiesis and pronounced germinal centre formation. By 16 days plasmacytosis was most marked in the spleens of mice bearing C-1 and was clearly apparent in mice bearing C-9, A-1 and H-1. In the spleens of mice bearing C-4 for 12 days, clear pink material appeared around the lymphoid sheaths. Thereafter there was progressive lymphoid atrophy, few lymphoid cells being seen at 22 days in the periarteriolar lymphoid sheath or elsewhere. This material did not stain with Congo Red.

*Lymph node histology*

All nodes draining sites of primary isografts showed a progressive increase in size. The axillary and inguinal nodes of mice bearing primary s.c. isografts of C-4, C-9 or H-1 all showed an increase in the size of the paracortical areas at 7 days, the

FIG. 2.—Splenomegaly in tumour-bearing mice. The vertical lines indicate standard errors.

cells therein being enlarged. From 12 days onwards plasmacytopoiesis, plasmacytosis and germinal centre formation were very prominent. In the nodes of mice bearing C-1 these latter changes were also marked, but changes in the paracortical areas were not apparent. The nodes of mice bearing C-1 showed tumour cell deposits by 12 days.

*Lactic dehydrogenase*

LDH activity was not elevated in the serum of mice bearing the tumours used here.

DISCUSSION

The experiments reported here provided evidence that, in mice bearing isografts of methylcholanthrene-induced sarcomas: (1) macrophages were stimulated; (2) the histological changes in lymph nodes indicated the occurrence of cell-mediated immune responses to tumours inducing concomitant immunity, but not to a tumour that failed to do so; (3) a variety of histological changes occurred in the spleens, the changes being different with different tumours but usually including increased haemopoiesis.

Stimulation of macrophages appears to be a common consequence of the growth of syngeneic tumours. The observations recorded here may be added to those by Blamey *et al.* (1969) on the increased phagocytic activity of the " reticulo-endothelial system " in tumour-bearing rats, and by Fisher *et al.* (1974) on increased monocytopoiesis in tumour-bearing mice. As macrophages activated in various ways have anti-tumour activity (Hibbs, 1973; Keller, 1973; Evans and Alexander, 1972, 1976) it should not be surprising if the converse were true and tumour immunity were accompanied by macrophage activation. A DNA synthetic response of macrophages in the unstimulated peritoneal cavity has been found to accompany cell-mediated immune reactions (More *et al.*, 1973) though it is not an inevitable accompaniment of activation, in the sense of intracellular microbicidal activity (North, 1970;

Nelson, 1972). It remains to be determined whether tumour growth brings about activation in this strict sense. The DNA-synthesizing cells detected by this method have been characterized as macrophages on the basis of their phagocytic capacity and adhesiveness. Those responding by increased DNA synthesis to soluble antigens injected into immunized mice have been shown to be previously resident in the peritoneal cavity (More *et al.*, 1973). It is possible that, in tumour-bearing mice, newly produced macrophages, recently arrived in the peritoneal cavity, contributed to the increased activity. In either case, however, the changes obviously reflect stimulation of some element of the mononuclear phagocytic system.

Macrophages proliferated in mice bearing the C-1 tumour, which failed to induce concomitant immunity and which metastasized. This may have occurred in association with a cell-mediated immune response of which other signs (*e.g.* histological) were not detected; or it may have occurred in the absence of a response by T cells, as can happen with *Corynebacterium parvum* (Woodruff, Dunbar and Ghaffar, 1973) and BCG (Pimm and Baldwin, 1975). A close correlation between macrophage activity and resistance to metastasis was found by Eccles and Alexander (1974) studying the macrophage content of rat tumours.

All the tumours studied evoked histological signs of immune responses in the regional lymph nodes, the spleen or both. The changes in lymph nodes were similar to those observed by others using 4 methylcholanthrene-induced sarcomas (Rosenau and Moon, 1966; Krüger, 1967), a mammary adenocarcinoma (Edwards *et al.*, 1971), and a lymphoma (Jurin and Drewinko, 1974). The strongest correlation with the development of concomitant tumour immunity was seen in the evocation of histological signs of cell-mediated immune responses by those tumours that induced concomitant immunity but not by the other tumour, C-1.

There are also parallels between the histological changes and the anti-tumour activity of spleen cells *in vivo* and *in vitro*. Spleen cells of mice bearing C-9 or H-1 are cytotoxic for target tumour cells *in vitro*. The cytotoxicity parallels the development of concomitant immunity and of histological changes; at first it is individual specific and involves T cells; later it is directed against other methylcholanthrene sarcoma cells and involves B cells (Kearney and Nelson, 1973; Kearney *et al.*, 1975). Spleen cells from mice bearing C-4 or A-1 were not cytotoxic *in vitro* or tumour suppressive *in vivo* (Simes *et al.*, 1975, and unpublished). This is clearly attributable to the lack of an immune response in the spleen and/or to dilution of immunologically active cells by non-lymphoid haemopoietic cells. Increased erythropoiesis has been known for some time to occur in tumour-bearing mice (Lockner, Sletten and De Hevesy, 1963; Edwards *et al.*, 1971). The lymphoid atrophy seen in the spleens of mice bearing C-4 is reminiscent of that caused by LDH-elevating virus (Snodgrass, Lowrey and Hanna, 1972) but there was no evidence of such an infection.

*Note added in proof*

It has recently been reported that the antibacterial activity of macrophages of mice bearing certain syngeneic tumours was, for a short time, depressed, after which it was enhanced; in the latter phase the mice exhibited concomitant tumour immunity (North, Kirstein and Tuttle, 1976).

We thank Maria van Deventer and Jean Penrose for skilled assistance. This work was supported in part by the New South Wales State Cancer Council, the Cancer Research Committee of The University of Sydney and the National Health and Medical Research Council. It was carried out in part pursuant to Research Contract NO1-CB-63973 with the United States National Cancer Institute.

### REFERENCES

BLAMEY, R. W., CROSBY, D. L. & BAKER, J. M. (1969) Reticuloendothelial Activity during the Growth of Rat Sarcomas. *Cancer Res.*, **29**, 335.

BLOOM, E. T. (1970) Quantitative Detection of Cytotoxic Antibodies against Tumor-specific Antigens of Murine Sarcomas Induced by 3-Methylcholanthrene. *J. natn. Cancer Inst.*, **45**, 443.

ECCLES, S. C. & ALEXANDER, P. (1974) Macrophage Content of Tumours in Relation to Metastatic Spread and Host Immune Reaction. *Nature, Lond.*, **250**, 667.

EDWARDS, A. J., SUMNER, M. R., ROWLAND, G. F. & HURD, C. M. (1971) Changes in Lymphoreticular Tissues during Growth of a Murine Adenocarcinoma. I. Histology and Weight of Lymph Nodes, Spleen and Thymus. *J. natn. Cancer Inst.*, **47**, 31.

EVANS, R. & ALEXANDER, P. (1972) Mechanism of Immunologically Specific Killing of Tumour Cells by Macrophages. *Nature, Lond.*, **236**, 168.

EVANS, R. & ALEXANDER, P. (1976) Mechanisms of Extracellular Killing of Nucleated Mammalian Cells by Macrophages. In *Immunobiology of the Macrophage*. Ed. D. S. Nelson. New York: Academic Press. p. 535.

FISHER, B., TAYLOR, S., LEVINE, M., SAFFER, E. & FISHER, E. R. (1974) Effect of *Mycobacterium bovis* (Strain *Bacillus Calmette-Guerin*) on Macrophage Production by the Bone Marrow of Tumour-bearing Mice. *Cancer Res.*, **34**, 1668.

HIBBS, J. B., JR (1973) Macrophage Nonimmunologic Recognition: Target Cell Factors Related to Contact Inhibition. *Science, N.Y.*, **180**, 868.

IZUMI, S., PENROSE, J. M., MORE, D. G. & NELSON, D. S. (1975) Further Observations on the Immunological Induction of DNA Synthesis in Mouse Peritoneal Macrophages. *Int. Archs Allergy appl. Immun.*, **49**, 573.

JURIN, M. & DREWINKO, B. (1974) Natural History of Mouse Syngeneic Lymphoma. *Am. J. Path.*, **77**, 213.

KEARNEY, R. & NELSON, D. S. (1973) Concomitant Immunity to Syngeneic Methylcholanthrene-induced Tumours in Mice. Occurrence and Specificity of Concomitant Immunity. *Aust. J. exp. Biol. med. Sci.*, **51**, 723.

KEARNEY, R., BASTEN, A. & NELSON, D. S. (1975) Cellular Basis for the Immune Response to Methylcholanthrene-induced Tumors in Mice. Heterogeneity of Effector Cells. *Int. J. Cancer*, **15**, 438.

KELLER, R. (1973) Cytostatic Elimination of Syngeneic Rat Tumor Cells *in vitro* by non-specifically Activated Macrophages. *J. exp. Med.*, **138**, 625.

KONDA, S. & SMITH, R. T. (1973) The Effects of Tumor Bearing upon Changes in Cell Distribution and Membrane Antigen Characteristics in Murine Spleen and Thymus Cell Subpopulations. *Cancer Res.*, **33**, 1878.

KONDA, S., NAKAO, Y. & SMITH, R. T. (1973) The Stimulatory Effect of Tumor Bearing upon the T- and B-cell Subpopulations of the Mouse Spleen. *Cancer Res.*, **33**, 2247.

KRÜGER, G. (1967) Morphologic Studies of Lymphoid Tissues during the Growth of an Isotransplanted Mouse Tumor. *J. natn. Cancer Inst.*, **39**, 1.

LOCKNER, D., SLETTEN, K. & DE HEVESY, G. (1963) Studies on Cancer Anaemia: Organ Weights, Blood Values and Iron Metabolism in Normal and Tumour-bearing Mice. *Br. J. Cancer*, **17**, 328.

MAGAREY, C. J. & BAUM, M. (1970) Reticulo-endothelial Activity in Humans with Cancer. *Br. J. Surg.*, **57**, 748.

MORE, D. G., PENROSE, J. M., KEARNEY, R. & NELSON, D. S. (1973) Immunological Induction of DNA Synthesis in Mouse Peritoneal Macrophages. *Int. Archs Allergy appl. Immun.*, **44**, 611.

NELSON, D. S. (1972) Macrophages as Effectors of Cellular Immunity. *CRC Crit. Rev. Microbiol.*, **1**, 353.

NELSON, D. S. (1974) Immunity to Infection, Allograft Immunity and Tumour Immunity: Parallels and Contrasts. *Transplant. Rev.*, **19**, 226.

NORTH, R. J. (1970) The Relative Importance of Blood Monocytes and Fixed Macrophages to the Expression of Cell-mediated Immunity to Infection. *J. exp. Med.*, **132**, 521.

NORTH, R. J., KIRSTEIN, D. P. & TUTTLE, R. L. (1976) Subversion of Host Defence Mechanisms by Murine Tumors. II. Counter-influence of Concomitant Antitumor Immunity. *J. exp. Med.*, **143**, 574.

PIMM, M. V. & BALDWIN, R. W. (1975) BCG Immunotherapy of Rat Tumours in Athymic Nude Mice. *Nature, Lond.*, **254**, 77.

ROSENAU, W. & MOON, H. D. (1966) Cellular Reactions to Methylcholanthrene-induced Sarcomas Transplanted to Isogenic Mice. *Lab. Invest.*, **15**, 1212.

SIMES, R. J., KEARNEY, R. & NELSON, D. S. (1975) Role of a Non-committed Accessory Cell in the *in vivo* Suppression of a Syngeneic Tumour by Immune Lymphocytes. *Immunology*, **29**, 343.

SMITH, R. T. & KONDA, S. (1973) The Stimulatory Effects of Bearing Primary Methylcholanthrene-induced Tumors upon the Murine Lymphoreticular System. *Int. J. Cancer*, **12**, 577.

SNODGRASS, M. J., LOWREY, D. S. & HANNA, M. G., JR. (1972) Changes Induced by Lactic Dehydrogenase Virus in Thymus and Thymus-dependent Areas of Lymphatic Tissue. *J. Immunol.*, **108**, 877.

WOODRUFF, M., DUNBAR, N. & GHAFFAR, A. (1973) The Growth of Tumours in T-cell Deprived Mice and their Response to Treatment with *Corynebacterium parvum*. *Proc. R. Soc.* B, **184**, 97.